# Rapid Identification of Geographical Origin of Commercial Soybean Marketed in Vietnam by ICP-MS

**DOI:** 10.1155/2021/5583860

**Published:** 2021-10-30

**Authors:** Trung Nguyen-Quang, Minh Bui-Quang, Minh Truong-Ngoc

**Affiliations:** ^1^Vietnam Academy of Science and Technology (VAST), Center for Research and Technology Transfer (CRETECH), 18 Hoang Quoc Viet Road, 100000 Hanoi, Vietnam; ^2^Vietnam Academy of Science and Technology (VAST), Graduate University of Science and Technology (GUST), 18 Hoang Quoc Viet Road, 100000 Hanoi, Vietnam

## Abstract

Inductively coupled plasma mass spectrometry (ICP-MS) analytical method was used to determine the content of 40 elements in 38 soybean samples (*Glycine Max*) from 4 countries. Multivariate statistical methods, such as principal components analysis (PCA), were performed to analyze the obtained data to establish the provenance of the soybeans. Although soybean is widely marketed in many countries, no universal method is used to discriminate the origin of these cereals. Our study introduced the initial step to the identification of the geographical origin of commercial soybean marketed in Vietnam. The analysis pointed out that there are significant differences in the mean of 33 of the 40 analyzed elements among 4 countries' soybean samples, namely, ^11^B, ^27^Al, ^44^Ca, ^45^Sc, ^47^Ti, ^55^Mn, ^56^Fe, ^59^Co, ^60^Ni, ^63^Cu, ^66^Zn, ^69^Ga, ^75^As, ^78^Se, ^85^Rb, ^88^Sr, ^89^Y, ^90^Zr, ^93^Nb, ^95^Mo, ^103^Rh, ^137^Ba, ^163^Dy, ^165^Ho, ^175^Lu, ^178^Hf, ^181^Ta, ^182^W, ^185^Re, ^197^Au, ^202^Hg, ^205^Tl, and ^208^Pb. The PCA analysis showed that the soybean samples can be classified correctly according to their original locations. This research can be used as a prerequisite for future studies of using the combination of elemental composition analysis with statistical classification methods for an accurate provenance establishment of soybean, which determined a variation of key markers for the original discrimination of soybean.

## 1. Introduction

In the last few years, numerous advancement in food authentication by using fingerprinting techniques has been reported [1–4], especially in the case of provenance determination. The majority of the methods are based on the combination of an analytical technique and one or multiple multivariate statistical analysis. First, the samples would be analyzed by a suitable analytical technique to acquire the data of interest, mostly tracing elements content or isotope ratio. Then, this data will be inspected by multivariate statistical analysis [2, 3] to gather the identification or categorization of the studied agriculture products in accordance with its geographical origin. This method relies on the assumption that the composition of an agricultural product's provenance soil will be reflected on the chemical composition of that product, such as wine [4–11], coffee [12], tea [13], olive oil, or fruit juice [14, 15], at least for some certain elements [14–16]. To ensure the success of this technique, suitable elements or isotopes must be selected carefully so that the soil geochemistry can be reflected by the chosen chemical, and from that, the products can be discriminated correctly. Only a few of the elements can satisfy the mentioned requirement. In addition, solid information of the element component in the sample, mostly at a trace level, is a must if this method would be applied at any degree of success. The most suitable technique for this purpose is inductively coupled plasma mass spectrometry (ICP-MS), with the ability to determine multiple elements in the sample [4–10].

Furthermore, the most common techniques used for food authenticity and traceability include isotope ratio; liquid and gas chromatography; elemental analysis, spectroscopic techniques, DNA-based techniques, and sensor techniques [17]. Spectroscopic techniques include vibrational [18], hyperspectral [19], fluorescence, and nuclear magnetic resonance [20]; these techniques are rapid and cost-effective and involve less or no sample preparation [21]. For example, Raman spectroscopy combined with support vector machine has been used to identify the rice-producing areas in China [22], with the correct rate, which was nearly 90%, and near-infrared spectroscopic technology combined with multivariate analysis. However, the main drawback is low accuracy due to less sensitivity and high noise.

In previous work, Yuji et al. [16] successfully distinguished the Japanese soybean from the one (*Glycine Max*) in China and classified the soybean between the interregional of Japan by using ICP-MS analysis combining with LDA model of 6 selected elements from the 24 elements with the use of backward stepwise regression, in particular Ba, Ca, Mn, Nd, W, and Ni. Besides, a commercial energy dispersion X-ray fluorescence (ED-XRF) was able to successfully measure 9 elements (Mg, K, Ca, Mn, Fe, Ni, Cu, Zn, and Rb) in 296 soybean samples from 5 producing areas of northern China (Henan, Inner Mongolia, Xinjiang, Heilongjiang, and Liaoning). The combination of MLP and ED-XRF overcomes the analytical disadvantages found with ICP-MS providing a novel and fast testing method which demonstrated to have a powerful classification capacity with an accuracy rate of 96.2% [12].

In Vietnam, the soybean planting area is not stable; domestic soybean production is only enough to supply about 8–10% of demand, which is up to nearly 200,000 tons/year. But due to the high demand, the import is up to more than 1 million tons/year, which is much higher than the number of domestic production. Similar to Japan and Korea, the lack of strict regulations on the management of agricultural products has led to a situation that people adulterate fake products with the authentic one to improve profits. Besides, they only mention the application of information technology on food traceability instead of the identification of geographical origins by chemical methods. Therefore, to learn from the experience of many countries in the world, the Vietnamese government will certainly need to review and amend the regulations to build geographical tracing methods based on chemical methods.

The difference in the elemental content of soybean samples is related to the content in soil and this is the key point to distinguish the geographical origin [23]. The growth and quality of soybeans are significantly affected by inorganic elements; for example, selenate at low concentrations (0.07 to 0.20 mg Se per kg seed) could promote the growth of soybeans and reduce cadmium [24]. The use of organic fertilizers and soil improvers, such as leonardite, might enrich the contents of macronutrients (Mg, Ca, K, and S) and micronutrients (Fe, Cu, Mn, and Zn).

In this study, the trace element composition of soybeans from 4 countries was compared to identify and classify them according to their origin. The result has shown that fingerprinting is a very promising method in collecting data to ascertain the soybeans' origin. Previous studies have pointed out these factors: the anthropogenic factors like the consumption of fertilizers and pesticides [6] or pollution [25] and natural factors such as heavy rains during the growing season or irrigation water. This study reported a new approach to originally discriminate the domestic and other imported soybeans in the Vietnam food market by using a combination of ICP-MS analysis and chemometric methods. This approach has been utilized to classify medicinal plants [10] or some types of foods and drinks, for example, tea [26], potato [27], wine [28], and honey [29], because of its high accuracy and sensitivity.

## 2. Materials and Methods

### 2.1. Materials

Thirty-eight soybean samples (15 from Vietnam, 8 from Canada, 9 from the US, and 6 from Brazil) packed in 2019 (Table 1) were used. Soybeans VN01-VN09 were provided by 9 supermarkets markets in Vietnam, whereas Soybeans VN10-VN15 were obtained from large residential Vietnamese's markets. Samples of imported soybeans (Can01-Can08, US01-US09, and Bra01-Bra06) were also provided by supermarkets in Vietnam. All of Vietnam samples came from supermarket and public market in Hanoi, Hai Phong originated from Vietnam northern regions or local farms (Ha Giang and Hanoi); public market in Da Nang, Can Tho, Saigon market; and public market in Ho Chi Minh City having soybeans from Dong Nai. Samples imported from Brazil were all originated from Mato Grosso; Canada samples from Can01 to Can 05 were from Ontario and Can 06 to Can 08 from Manitoba; US samples US01 and US04 originated from Iowa, and other samples came from Illinois. All of the collected samples were stored at −20°C in a deep freezer before they were analyzed.

### 2.2. Chemicals and Reagents

Nitric acid 65% (HNO_3_) and hydrogen peroxide 30% (H_2_O_2_) solutions were purchased from Merck, USA. Ultrapure deionized water with a resistivity of 18.2 MΩ-cm was obtained from a Milli-Q Plus water purification system (Millipore, Bedford, MA, USA). Twenty-one multielement standard solutions including ^11^B, ^27^Al, ^44^Ca, ^55^Mn, ^56^Fe, ^59^Co, ^60^Ni, ^63^Cu, ^66^Zn, ^69^Ga, ^75^As, ^78^Se, ^85^Rb, ^88^Sr, ^137^Ba, ^197^Au, ^202^Hg, ^205^Tl, ^208^Pb, ^24^Mg, and ^28^Si (10mg/L each element) (TraceCERT, periodic table mix 1 for ICP, product no. 92091, Lot: BCBW5563) and eight rare-earth elements (^45^Sc, ^89^Y, ^163^Dy, ^165^Ho, ^139^La, ^159^Tb, ^169^Tm, and ^175^Lu, 10 mg/L each element) were provided by Sigma-Aldrich Company. A standard solution containing 50 *µ*g/L of ^47^Ti, ^90^Zr, ^93^Nb, ^95^Mo, ^103^Rh, ^178^Hf, ^181^Ta, ^182^W, ^185^Re, ^232^Th, and ^238^U in 1% HNO_3_ was used to determine the sensitivity factors for all elements across the entire mass range for the measurement of diluted samples made in semiquantitative mode. If digested samples were analyzed, ethanol would be excluded from the calibration solution. Meanwhile, analysis grade ethanol was used for preparing matrix-matched standards. The internal standard (^45^Sc, ^49^In, ^83^Bi, ^89^Y, ^159^Tb, and ^32^Ge) for the quantitative analysis would be made in 1% HNO_3_ for the diluted sample, and only in 0.14 M HNO_3_ for the digested one. Similarly, a solution of in 1% HNO_3_ and only 0.14 M HNO_3_ containing 50 *µ*g/L of the internal standard would be used as the blank for the diluted and digested sample analysis in that order. Both standard and internal standard in this studied were prepared by diluting the 1000 mg/L standard stock solution.

### 2.3. Sample Preparation and ICP-MS Measurements

Accurately weigh 0.5 g of each soybean sample to be placed in a Teflon tube, and then add into the tube 4 mL of concentrated HNO_3_ (Merck, Germany) and 1 mL of 30% H_2_O_2_ (Merck, Germany). Next, transfer the tubes to the microwave oven MARS6 (CEM, US) with the following setting power: 1000–1800 W and temperature: 190°C for 20 minutes. The samples (25 mL) were cooled to room temperature and then diluted with deionized water up to the mark before being analyzed on an Agilent 7900 ICP-MS system (Agilent, Japan). The standard curve was built using the ICP multielemental standard solution at six concentrations 1.0; 2.0; 5.0; 10.0; 20.0; and 50.0 *μ*g/L. The content of each element was calculated based on the standard curves established under the same conditions [30–35]. An Agilent 7900 ICP-MS instrument (Agilent Technologies, Tokyo, Japan) was utilized for the measurement of 40 elements in the soybean samples, which were ^11^B, ^24^Mg, ^27^Al, ^28^Si, ^44^Ca, ^45^Sc, ^47^Ti, ^55^Mn, ^56^Fe, ^59^Co, ^60^Ni, ^63^Cu, ^66^Zn, ^69^Ga, ^75^As, ^78^Se, ^85^Rb, ^88^Sr, ^89^Y, ^90^Zr, ^93^Nb, ^95^Mo, ^103^Rh, ^137^Ba, ^139^La, ^159^Tb, ^163^Dy, ^165^Ho, ^169^Tm, ^175^Lu, ^178^Hf, ^181^Ta, ^182^W, ^185^Re ^197^Au, ^202^Hg, ^205^Tl, ^208^Pb, ^232^Th, and ^238^U. The analytical parameters of the ICP-MS were RF power at 1550 W, RF matching at 2.0 V, cell entrance at −40 V, cell exit of −60 V, cell energy discrimination at 5.0 V, and spray chamber temperature at 2°C. Argon was used as carrier gas at flowrate 1.09 L/min, and Helium was used to eliminate interferences at 4.3 L/min. Data quantitation was achieved regarding matrix-matched multielement standards that had been prepared in 1% HNO_3_ [35–38].

### 2.4. Method Validation

In this study, instrument detection limits were calculated using the raw intensity data from the standard and the blank (using ultrapure 2% nitric acid matrix) as per the following equation: IDL = 3SD_blank_ × *C*_*x*_/(*S*_*x*_ − *S*_blank_), where SD_blank_ is the standard deviation of the intensities of the multiple blank measurements, *C*_*x*_ is the mean signal for the standard, and then *S*_*x*_ is signal for *C*_*x*_ and *S*_blank_ is signal for blank. Method detection limits (MDLs) were calculated as follows: MDL = IDL × constant volume/sample weight.

Calibration verification standards were prepared from single element ICP standards (Merck) consisting of 3 different sets: Ca, Mg for the high standard series and Al, B, Cu, Rb, Sr, Zn and Cd, Co, Cs, Ni, Tl, V for the low standard series. The calibration verifications were measured after every 10 samples.

The duplicate of two soybean samples was made. Interferences from matrix were examined by evaluating an interference check sample composed of ^56^Fe, Ca, ^63^Cu, and ^66^Zn. Besides, serial dilutions and spike recovery tests were performed with the soybean samples. The serial dilution check was tested by diluting 1 : 10 and then 1 : 3 (thus the final dilution is 1 : 30) with one sample. Several elements were spiked to the soybean samples at the concentration level of 20 and 100 *µ*g/L for ^27^Al, ^63^Cu, and ^88^Sr and 100 and 500 *µ*g/L for the elements ^11^B, ^55^Mn, ^66^Zn, and ^85^Rb [39].

### 2.5. Statistical Analysis

Data acquisition and processing were performed by Microsoft Excel 2016 (Microsoft Corporation, USA). For normalization of data, each value of an elemental content was divided by the difference of maximum and minimum contents of the element among samples. The principal component analysis (PCA) was performed on the STATISTICA 12 (Dell Software, USA) and hierarchical clustering analysis (HCA) was implemented on the R package (R Foundation for Statistical Computing, Vienna, Austria).

## 3. Results and Discussion

### 3.1. Selection of Elements for Multivariate Analysis

Recently, the public has paid significant attention to the toxicity of potentially harmful chemical substances contained in food [40, 41]. These compounds could cause consequential negative effects on human health, such as food poisoning or cancer. As the result, there is an increase in demand for performing scientific studies in this field in order to extend our knowledge about the impact of the hazardous components in our daily food [42–44]. Among the daily food, soybean is one of the most frequently studied subjects, which mostly focuses on the composition of heavy metals (such as ^75^As, ^63^Cu, ^48^Cd, and ^208^Pb), other inorganic compounds, and organic substances [45–49]. Beside soybean safety consumption limit study, this material could also be utilized for other approaches, such as fertilizer residues or polyphenols [50–53]. Based on previous studies, it can be concluded that the origin of samples, also known as the history of the product, can be explicated by analyzing the composition of trace elements [10, 54–57]. This is especially true with the soybean matrix, as the soybean sample is relatively homogeneous. Besides, collecting soybean in a large number of sample representatives for a large area is a possible and quite easy task.

The results for the analysis of the soybean samples are summarized in Table 2 for the 40 elements (^11^B, ^14^Si, ^24^Mg, ^27^Al, ^44^Ca, ^45^Sc, ^47^Ti, ^55^Mn, ^56^Fe, ^59^Co, ^60^Ni, ^63^Cu, ^66^Zn, ^69^Ga, ^75^As, ^78^Se, ^85^Rb, ^88^Sr, ^89^Y, ^90^Zr, ^93^Nb, ^95^Mo, ^103^Rh, ^137^Ba, ^139^La, ^159^Tb, ^163^Dy, ^165^Ho, ^169^Tm, ^175^Lu, ^178^Hf, ^181^Ta, ^182^W, ^185^Re, ^197^Au, ^202^Hg, ^205^Tl, ^208^Pb, ^232^Th, and ^238^U).

To verify the measurement results, the data were compared with black soybean data [16]. Since these are two different types of soybean, there were several differences regarding the mineral absorbed by the plant and the concentration of the minerals. There were 13 elements shared between two data sets, in which 12 had their data since the ^24^Mg measure was lower than the method detection limit of this experiment. The concentrations of Ca from the four countries were lower than the black soybean from Japan, the highest only 908 *µ*g/g compared to 1400 *µ*g/g of Japan black soybean. The concentrations of ^55^Mn and ^182^W were 2 to 4 times higher than the concentration of those in Japan black soybean. The concentration of the other elements had mixed measurement; some soybean countries had a certain element concentration higher and some lower than Japan black soybean. These results show that the method results were reliable and suitable for further analysis.

There are overlaps in the concentration range of most elements within the four regions. However, the concentration level of these elements still can be inferred based on the variation of each element concentration level in each region. An examination was done with various binary and ternary scatterplots from different element combinations. In general, multiple combinations of several elements could sufficiently distinguish between any two of the regions. However, it is not enough if a classification for all four regions is required. Thus, the use of scatterplot is not adequate to clarify the differences for the categorization within the four groups. Since the sample size, in this case is the number of soybean samples, was relatively small compared with the number of variables (the number of analyzed element concentration), reducing the number of variables is essential to be able to effectively conduct multivariate statistical analysis. A significant difference of group means at the confidence level of 95% was found by using ANOVA test for these following elements: ^11^B, ^27^Al, ^44^Ca, ^45^Sc, ^47^Ti, ^55^Mn, ^56^Fe, ^59^Co, ^60^Ni, ^63^Cu, ^66^Zn, ^69^Ga, ^75^As, ^78^Se, ^85^Rb, ^88^Sr, ^89^Y, ^90^Zr, ^93^Nb, ^95^Mo, ^103^Rh, ^137^Ba, ^163^Dy, ^165^Ho, ^175^Lu, ^178^Hf, ^181^Ta, ^182^W, ^185^Re, ^197^Au, ^202^Hg, ^205^Tl, and ^208^Pb. Besides, a few elements (^139^La, ^24^Mg, ^24^Si, and ^238^U) were removed due to the large analytical uncertainty. There are two reasons for this: a high polyatomic background interference, and the element's concentration levels being close to the MDL of the method.

The ICP-MS results for the samples, which were shown in Table S1, indicated that the contents of heavy metals (^75^As, ^63^Cu, ^48^Cd, ^208^Pb) and toxic metals (^137^Ba, ^205^Tl) in all testing samples were lower than the limiting standards according to the Ministry of Health of Vietnam (0.1 mg/kg for ^48^Cd, and 0.2 mg/kg for ^208^Pb) [58]. Thus, these samples met the demands of manufacturing in the Vietnamese food market. Then, the ICP-MS data were used for further multivariate statistics.

### 3.2. Geographically Original Discrimination of Soybeans

**Figure 1:**
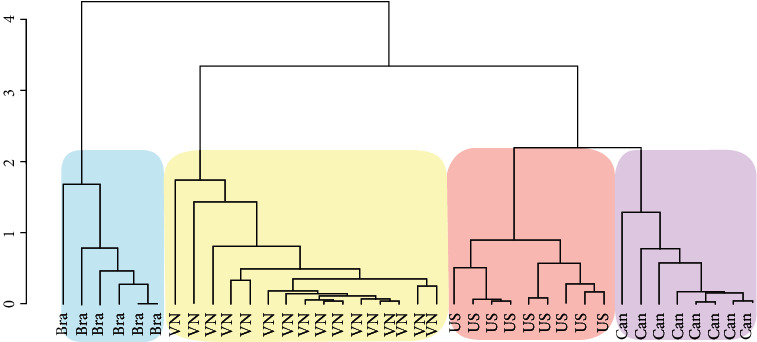
HCA dendrogram of soybean samples.

Since the values of the contents were thousandfold different among elements, a min-max normalization method was applied to make all values between 0 and 1. By doing so, all elemental values were standardized into a common scale. In detail, the difference between an elemental content of a sample and the minimum content of this element among samples was divided by the distance between the highest and lowest values of the element. The normalized data were analyzed by multivariate statistical methods, such as HCA and PCA to reduce the data dimension and supply insight discrimination of the samples. On the one hand, the HCA model classified samples by measuring similarities through non-Euclidean distance, which was performed in Figure 1. As can be seen, 38 samples were sharply clustered into four groups based on their origins. While Canadian and US samples had a gentle correlation, the dendrogram witnessed the dramatic separation of soybeans from Vietnam and Brazil.

**Figure 2:**
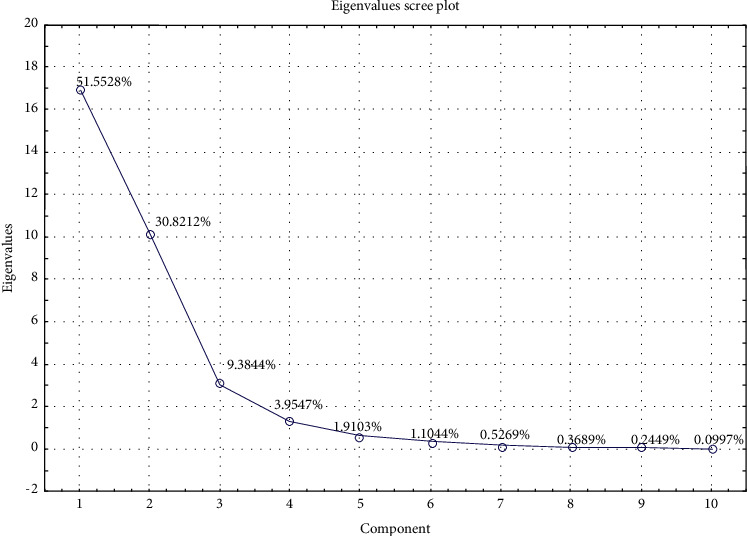
Eigenvalues scree plot.

On the other hand, the data set was further processed on the PCA models not only to distinguish soybean origins but also to identify key elements for the discrimination. From the scree plot of eigenvalues (Figure 2), the first three principal components (PCs) accounted for 90% of the total variance, where 51.6% and 30.8% of the sample variability were explained by PC1 and PC2, respectively.

**Figure 3:**
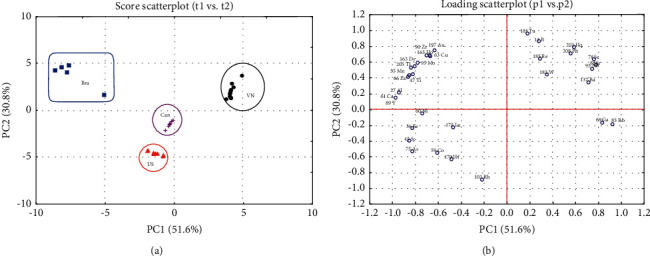
PCA score (a) and (b) loading plots of soybean samples. VN: Vietnamese samples; Can: Canadian samples; Br: Brazilian samples; US: the US samples.

Figure 3(a) illustrates the PCA score plot of 38 soybean samples, which were sharply separated by their geographical origins. On the loading plot (Figure 3(b)), variables with the highest absolute values in the vertical or horizontal axis had higher influences on the differentiation of the cases on the score plot. The result in Figure 3(b) showed that more elements had positive loadings on PC2 and negative loadings on PC1. ^69^Ga, ^85^Rb, and ^89^Y gave the highest contribution for the separation on PC1, while ^103^Rh and ^108^Ta had the strongest effect on PC2. In addition, the variables of which the position is represented on the loading plot similar to the position of the cases on the score plot will be the characteristic variables for that group of functions. In other words, an element will be the “key” for the classification of a certain sample group if their representations on the mentioned graphs are the same. As can be seen, Vietnamese soybeans were distinguished by positive PC1 and PC2. The loading plot indicated the positive values on both the first two PCs of ^78^Se, ^88^Sr, ^93^Nb, and ^137^Ba at similar positions of those samples on the score plot, which could be explained for the cluster of soybeans from Vietnam. In addition, X and moving R charts (Figures S1a–S1d) pointed out that soybeans from Vietnam had the highest contents of those four elements, compared to the importing samples.

Next, the significant separation of soybeans exporting from Brazil was affected by a variety of metals, such as ^47^Ti, ^55^Mn, ^66^Zn ^95^Mo, ^163^Dy, and ^205^Tl, since the content of these elements was considerably higher in Brazilian samples than in the other ones (Figures S2a–S2f). For example, ^47^Ti in Brazilian exporting soybeans ranged from 31 to 42 ppm, while the figures for samples from other sources were mostly under 30 ppm. Similarly, ^55^Mn, ^66^Zn ^95^Mo, and ^205^Tl contents in Brazilian soybeans might be at least 1.5 to 10 times higher than in other samples. Considerably, though found at a low concentration, ^163^Dy could be found only in Brazilian soybeans (Table S1).

Although clustering at nearby positions on the PCA score plot, Canadian and US samples could be discriminated by certain elements on the basis of the loading plot and the moving charts, as shown in Figure 3(b) and Figures S3 and S4. While the highest content among all samples of ^175^Lu in the Canadian soybeans might be the key for identification of this group (Figure S3 and Table S1), ^59^Ti and ^178^Hf were the markers to distinguish soybeans from the US due to the higher content of the elements in this sample group than the other ones (Figure S4 and Table S1). The nearby positions of those two clusters could be explained by the similar content of ^103^Rh (Figure S5) in both the US and Canadian soybeans. While the element had the strongest negative effect on the PC2, these two groups also shared the negative PC2 values.

Overall, both HCA and PCA methods illustrated the clustering of soybean samples based on their different geographical origins. Samples from Vietnam could be distinguished from other imported groups by the higher contents of ^78^Se, ^88^Sr, ^93^Nb, and ^137^Ba, whereas Brazilian soybeans could be classified based on several key elements, such as ^47^Ti, ^55^Mn, ^66^Zn ^95^Mo, ^163^Dy, and ^205^Tl. Meanwhile, the discrimination of the US and Canadian soybeans depended on the typical contents of ^175^Lu for samples from Canadian or ^59^Ti and ^178^Hf for samples from the US. To the best of our knowledge, this is the first study that discriminates soybeans in the Vietnam food market using ICP-MS-based metallomics approach.

## 4. Conclusions

This study documented that classifying soybean from 4 countries according to their geographical origin gives further evidence of the ability of multivariate statistical analysis based on trace element data to show provenance. The elemental contents of soybean from Vietnam were specific enough to be distinguished from imported types; meanwhile, the samples from Brazil, Canada, and the USA could be classified clearly. Therefore, the developed method for the determination of 33 elements by ICP-MS could be used for identifying the authenticity of soybeans according to geographical origin growing in Vietnam, as well as imported samples from other countries. It could be considered as a promising, rapid, and cost-effective method to evaluate soybean and other food origins.

## Figures and Tables

**Table 1:** Summary of soybean samples analyses throughout the study.

Country	Code	Region	Supermarkets
Vietnam	VN01	Ha Giang	AEON Vietnam
	VN02	Ha Giang	Big C
	VN03	Ha Giang	Co.op mart
	VN04	Ha Giang	Mega Market Vietnam
	VN05	Hanoi	Lotte Mart
	VN06	Dong Nai	Emart
	VN07	Hanoi	VinMart
	VN08	Dong Nai	LanChi Mart
	VN09	Hanoi	Fivimart
	VN10	Dong Nai	Saigon Market
	VN11	Hanoi	Public market in Hanoi
	VN12	Dong Nai	Public market in Ho Chi Minh City
	VN13	Ha Giang	Public market in Hai Phong
	VN14	Dong Nai	Public market in Da Nang
	VN15	Dong Nai	Public market in Can Tho
Canada	Can01	Ontario	Big C
	Can02	Ontario	Co.op mart
	Can03	Ontario	Mega Market Vietnam
	Can04	Ontario	Lotte Mart
	Can05	Ontario	Emart
	Can06	Manitoba	LanChi Mart
	Can07	Manitoba	Fivimart
	Can08	Manitoba	Saigon Market
United States	US01	Iowa	Big C
	US02	Iowa	Co.op mart
	US03	Iowa	Mega Market Vietnam
	US04	Iowa	Lotte Mart
	US05	Illinois	Emart
	US06	Illinois	VinMart
	US07	Illinois	LanChi Mart
	US08	Illinois	Fivimart
	US09	Illinois	Saigon Market
Brazil	Bra01	Mato Grosso	Big C
	Bra02	Mato Grosso	Co.op mart
	Bra03	Mato Grosso	Mega Market Vietnam
	Bra04	Mato Grosso	Lotte Mart
	Bra05	Mato Grosso	Emart
	Bra06	Mato Grosso	Fivimart

**Table 2:** Contents (*µ*g/g, fresh weight base) of the 40 elements in soybean from 4 countries.

Element	Region			
	Canada	US	Brazil	Vietnam
	(*n* = 8)	(*n* = 9)	(*n* = 6)	(*n* = 15)
^11^B	423.25 ± 16.851	336.8 ± 24.735	393.86 ± 61.80	483.93 ± 11.305
^28^Si	<MDL	<MDL	<MDL	<MDL
^24^Mg	<MDL	<MDL	<MDL	<MDL
^27^Al	840.93 ± 15.08	1363 ± 113.56	2379.3 ± 1103.3	535.27 ± 15.791
^43^Ca	614.16 ± 11.52	561.85 ± 41.81	908.07 ± 14.59	279.28 ± 6.78
^45^Sc	<MDL	<MDL	<MDL	<MDL
^47^Ti	27.253 ± 2.2199	25.813 ± 2.0944	32.832 ± 1.724	24.087 ± 0.5734
^55^Mn	45.866 ± 0.9681	45.897 ± 3.8135	53.095 ± 1.555	44.133 ± 1.0460
^56^Fe	31.988 ± 2.313	94.76 ± 7.07	13.542 ± 5.908	23.52 ± 2.11
^59^Co	0.0534 ± 0.0026	0.2290 ± 0.0196	0.1438 ± 0.0093	0.0467 ± 0.0011
^60^Ni	16.567 ± 0.3169	50.421 ± 3.8666	50.017 ± 1.466	25.14 ± 0.5817
^63^Cu	77.783 ± 7.7178	47.226 ± 3.9288	10.26 ± 0.4853	63.227 ± 1.4837
^66^Zn	58.743 ± 0.9605	46.583 ± 0.3609	73.325 ± 1.754	43.533 ± 1.0164
^69^Ga	8.6455 ± 1.075	14.67 ± 1.1573	5.2507 ± 3.9859	16.792 ± 0.3872
^75^As	0.342 ± 0.0118	0.4308 ± 0.038	0.3563 ± 0.0336	0.1385 ± 0.0031
^78^Se	2.4045 ± 0.1028	1.4091 ± 0.0047	1.8943 ± 0.0140	5.3848 ± 0.0231
^85^Rb	27.161 ± 0.2047	29.675 ± 0.2124	12.522 ± 0.7153	35.208 ± 0.8644
^88^Sr	0.97807 ± 0.031	0.82645 ± 0.020	1.0689 ± 0.047	5.2305 ± 0.0217
^89^Y	0.121 ± 0.0082	0.1456 ± 0.0124	0.2112 ± 0.0123	0.0716 ± 0.0017
^90^Zr	0.8295 ± 0.0639	0.4728 ± 0.0371	1.5277 ± 0.0205	0.7286 ± 0.0170
^93^Nb	0.0314 ± 0.0037	0.0395 ± 0.003	0.0349 ± 0.0033	0.0849 ± 0.0019
^95^Mo	4.9766 ± 0.2664	5.9762 ± 0.5723	38.284 ± 2.137	7.1016 ± 0.2418
^103^Rh	0.0017 ± 0.0008	0.0022 ± 0.0001	0.0002 ± 0.0007	<MDL
^137^Ba	0.61 ± 0.0019	0.704 ± 0.018	0.95 ± 0.047	4.205 ± 0.268
^139^La	<MDL	<MDL	<MDL	<MDL
^208^Pb	<MDL	<MDL	<MDL	<MDL
^163^Dy	<MDL	<MDL	0.0092 ± 0.0009	<MDL
^165^Ho	<MDL	<MDL	0.0017 ± 0.0001	0.3003 ± 0.0023
^69^Tm	<MDL	<MDL	<MDL	<MDL
^175^Lu	<MDL	<MDL	<MDL	<MDL
^178^Hf	0.0035 ± 0.0002	0.0127 ± 0.0009	0.0066 ± 0.0002	0.0024 ± 0.0002
^181^Ta	0.0582 ± 0.0046	0.0324 ± 0.0024	0.0637 ± 0.0005	0.0753 ± 0.0018
^182^W	0.01151 ± 0.001	0.01206 ± 0.001	0.01116 ± 0.004	0.01425 ± 0.001
^185^Re	0.1822 ± 0.0364	0.209 ± 0.0155	0.2216 ± 0.0082	0.2739 ± 0.0085
^197^Au	0.0049 ± 0.0002	0.0015 ± 0.0001	0.0448 ± 0.0076	0.0138 ± 0.0003
^202^Hg	0.0143 ± 0.0017	0.0058 ± 0.0004	0.0121 ± 0.0058	0.0232 ± 0.0005
^205^Tl	0.0022 ± 0.0001	<MDL	0.0016 ± 0.0001	<MDL
^208^Pb	0.0263 ± 0.0003	0.0079 ± 0.0006	0.0195 ± 0.0099	0.0396 ± 0.0020
^90^Th	<MDL	<MDL	<MDL	<MDL
^92^U	<MDL	<MDL	<MDL	<MDL

Data express means ± SD (standard deviation); MDL: method detection limit.

## Data Availability

The majority of the data used in this study are included in the article. Other data can be made available upon request from the corresponding author.
