# Supplementary material for: Rapid Identification of Geographical Origin of Commercial Soybean Marketed in Vietnam by ICP-MS
Source: J Anal Methods Chem. 2021 Oct 30;2021:5583860. doi: 10.1155/2021/5583860 (PMC8572128; doi:10.1155/2021/5583860)
Supplement: Supplementary Materials — Figures S1–S5 and Table S1 are provided. [file 5583860.f1.zip › 5583860.f1/Figure S3.docx]

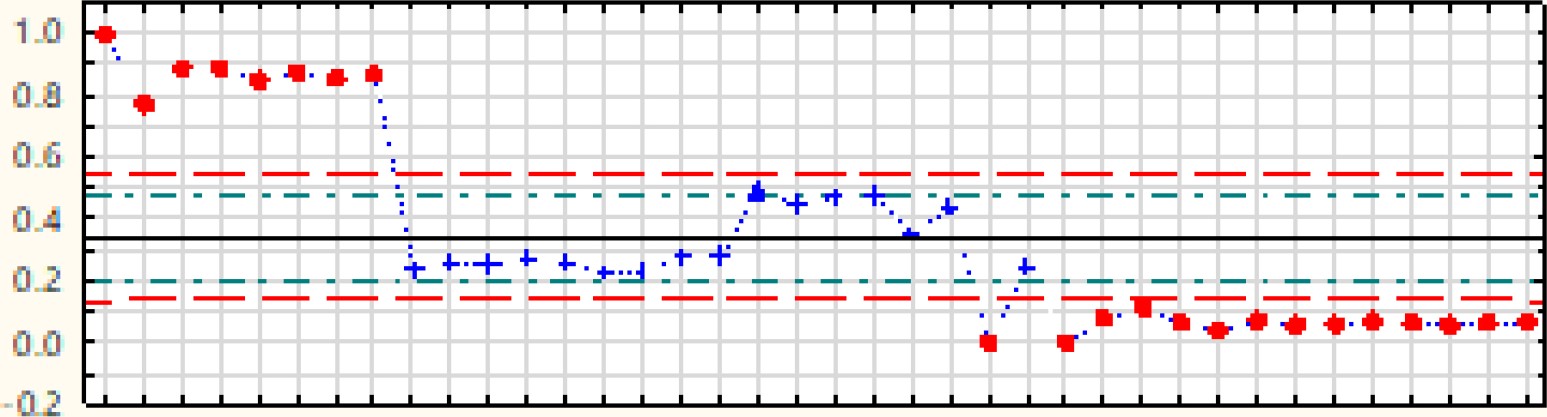


**Figure S3**. X and Moving R chart of ^175^Lu

0.135

0.204

0.343

0.481

0.550

X and Moving R Chart; variable: 175 Lu

Bra06

VN15

VN14

VN13

VN12

VN11

VN10

VN09

VN08

VN07

VN06

VN05

VN04

VN03

VN02

VN01

Bra05

Bra04

Bra03

Bra02

Bra01

US09

US08

US07

US06

US05

US04

US03

US02

US01

Can08

Can07

Can06

Can05

Can04

Can03

-0.2

0

0.2

Can02

Can01

0.4

1.2

1.0

0.8

0.6
