# Supplementary material for: Rapid Identification of Geographical Origin of Commercial Soybean Marketed in Vietnam by ICP-MS
Source: J Anal Methods Chem. 2021 Oct 30;2021:5583860. doi: 10.1155/2021/5583860 (PMC8572128; doi:10.1155/2021/5583860)

0.6

0.8

1.0

0.4

Can01

Can02

0.2

0

-0.2

Can03

Can04

Can05

Can06

Can07

Can08

US01

US02

US03

US04

US05

US06

US07

US08

US09

Bra01

Bra02

Bra03

Bra04

Bra05

VN01

VN02

VN03

VN04

VN05

VN06

VN07

VN08

VN09

VN10

VN11

VN12

VN13

VN14

VN15

Bra06

X and Moving R Chart; variable: 103 Rh

0.537

0.453

0.283

0.114

0.029

**Figure S5**. X and Moving R chart of ^103^Rh


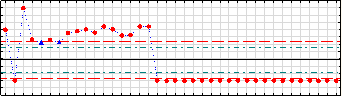

Supplement: Supplementary Materials — Figures S1–S5 and Table S1 are provided. [file 5583860.f1.zip › 5583860.f1/Figure S5.docx]
