# Supplementary material for: Rapid Identification of Geographical Origin of Commercial Soybean Marketed in Vietnam by ICP-MS
Source: J Anal Methods Chem. 2021 Oct 30;2021:5583860. doi: 10.1155/2021/5583860 (PMC8572128; doi:10.1155/2021/5583860)
Supplement: Supplementary Materials — Figures S1–S5 and Table S1 are provided. [file 5583860.f1.zip › 5583860.f1/Table S1 (1).docx]

**Table S1**. Contents (Mean±SD, µg/g) of the 33 elements in 38 soybean samples

| **Sample** | **^11^B** | **^27^Al** | **^45^Sc** | **^44^Ca** | **^47^Ti** | **^55^Mn** | **^56^Fe** | **^59^Co** | **^60^Ni** | **^63^Cu** | **^66^Zn** |
| --- | --- | --- | --- | --- | --- | --- | --- | --- | --- | --- | --- |
| Can01 | 507.0±6.4 | 1024.0±12.8 | 0.8772±0.0110 | 5.908±0.074 | 27.56±0.35 | 480.00±6.02 | 0.3883±0.0049 | 0.4745±0.0060 | 17.650±0.221 | 60.150±0.754 | 468.3±5.9 |
| Can02 | 383.0±4.8 | 523.2±6.6 | 0.8361±0.0105 | 6.114±0.077 | 22.89±0.29 | 457.00±5.73 | 0.3898±0.0049 | 0.5423±0.0068 | 18.570±0.233 | 85.740±1.075 | 570.2±7.2 |
| Can03 | 405.8±5.1 | 1014.0±12.7 | 0.7949±0.0100 | 6.319±0.079 | 31.07±0.39 | 446.30±5.60 | 0.3867±0.0048 | 0.5641±0.0071 | 14.020±0.176 | 81.940±1.028 | 680.8±8.5 |
| Can04 | 431.9±5.4 | 853.8±10.7 | 0.8361±0.0105 | 6.114±0.077 | 27.17±0.34 | 461.10±5.78 | 0.3883±0.0049 | 0.5270±0.0066 | 16.750±0.210 | 75.940±0.952 | 573.1±7.2 |
| Can05 | 406.9±5.1 | 797.0±10.0 | 0.8223±0.0103 | 6.182±0.078 | 27.04±0.34 | 454.80±5.70 | 0.3883±0.0049 | 0.5445±0.0068 | 16.450±0.206 | 81.200±1.018 | 608.0±7.6 |
| Can06 | 425.7±5.3 | 839.6±10.5 | 0.8326±0.0104 | 6.131±0.077 | 27.14±0.34 | 459.50±5.76 | 0.3883±0.0049 | 0.5314±0.0067 | 16.670±0.209 | 77.260±0.969 | 581.8±7.3 |
| Can07 | 411.6±5.2 | 807.7±10.1 | 0.8249±0.0103 | 6.169±0.077 | 27.07±0.34 | 456.00±5.72 | 0.3883±0.0049 | 0.5412±0.0068 | 16.500±0.207 | 80.220±1.006 | 601.5±7.5 |
| Can08 | 414.1±5.2 | 868.2±10.9 | 0.8196±0.0103 | 6.196±0.078 | 28.09±0.35 | 454.60±5.70 | 0.3879±0.0049 | 0.5442±0.0068 | 15.930±0.200 | 79.820±1.001 | 615.8±7.7 |
| US1 | 329.4±4.1 | 1438.0±18.0 | 0.8939±0.0112 | 5.200±0.065 | 26.75±0.34 | 483.50±6.06 | 0.6109±0.0077 | 2.4570±0.0308 | 49.660±0.623 | 49.810±0.625 | 498.2±6.2 |
| US2 | 324.0±4.1 | 1276.0±16.0 | 0.8554±0.0107 | 5.695±0.071 | 24.46±0.31 | 430.10±5.39 | 0.6623±0.0083 | 2.1180±0.0266 | 49.320±0.618 | 44.220±0.555 | 432.1±5.4 |
| US3 | 326.7±4.1 | 1357.0±17.0 | 0.8746±0.0110 | 5.448±0.068 | 25.60±0.32 | 456.80±5.73 | 0.6366±0.0080 | 2.2880±0.0287 | 49.490±0.621 | 47.020±0.590 | 465.1±5.8 |
| US4 | 358.6±4.5 | 1490.0±18.7 | 0.9602±0.0120 | 5.980±0.075 | 28.11±0.35 | 501.50±6.29 | 0.6989±0.0088 | 2.5120±0.0315 | 54.330±0.681 | 51.610±0.647 | 510.6±6.4 |
| US5 | 336.4±4.2 | 1374.0±17.2 | 0.8967±0.0112 | 5.708±0.072 | 26.06±0.33 | 462.80±5.80 | 0.6659±0.0084 | 2.3060±0.0289 | 51.050±0.640 | 47.620±0.597 | 469.3±5.9 |
| US6 | 296.5±3.7 | 1227.0±15.4 | 0.7930±0.0099 | 4.966±0.062 | 23.17±0.29 | 413.00±5.18 | 0.5801±0.0073 | 2.0660±0.0259 | 44.940±0.564 | 42.500±0.533 | 420.1±5.3 |
| US7 | 320.9±4.0 | 1220.0±15.3 | 0.7974±0.0100 | 5.087±0.064 | 23.15±0.29 | 411.00±5.15 | 0.5934±0.0074 | 2.0470±0.0257 | 45.420±0.570 | 42.290±0.530 | 416.6±5.2 |
| US8 | 365.9±4.6 | 1487.0±18.6 | 0.9620±0.0121 | 6.033±0.076 | 28.09±0.35 | 500.60±6.28 | 0.7047±0.0088 | 2.5030±0.0314 | 54.530±0.684 | 51.510±0.646 | 509.0±6.4 |
| US9 | 365.4±4.6 | 1473.0±18.5 | 0.9562±0.0120 | 6.031±0.076 | 27.87±0.35 | 496.00±6.22 | 0.7041±0.0088 | 2.4770±0.0311 | 54.290±0.681 | 51.040±0.640 | 503.9±6.3 |
| Bra1 | 522.3±6.5 | 3317.0±41.6 | 0.9084±0.0114 | 10.530±0.132 | 39.13±0.49 | 612.10±7.68 | 0.7261±0.0091 | 1.4070±0.0176 | 59.970±0.752 | 125.000±1.568 | 976.3±12.2 |
| Bra2 | 457.1±5.7 | 2850.0±35.7 | 0.9730±0.0122 | 12.690±0.159 | 42.88±0.54 | 697.10±8.74 | 0.6945±0.0087 | 1.7340±0.0217 | 61.060±0.766 | 137.300±1.722 | 903.6±11.3 |
| Bra3 | 489.7±6.1 | 3084.0±38.7 | 0.9407±0.0118 | 11.610±0.146 | 41.01±0.51 | 654.60±8.21 | 0.7103±0.0089 | 1.5710±0.0197 | 60.510±0.759 | 131.200±1.645 | 939.9±11.8 |
| Bra4 | 489.7±6.1 | 3084.0±38.7 | 0.9407±0.0118 | 11.610±0.146 | 41.01±0.51 | 654.60±8.21 | 0.7103±0.0089 | 1.5710±0.0197 | 60.510±0.759 | 131.200±1.645 | 939.9±11.8 |
| Bra5 | 370.7±4.6 | 2334.0±29.3 | 0.7121±0.0089 | 8.789±0.110 | 31.04±0.39 | 495.50±6.21 | 0.5377±0.0067 | 1.1890±0.0149 | 45.810±0.574 | 99.290±1.245 | 711.5±8.9 |
| Bra6 | 459.9±5.8 | 2889.0±36.2 | 0.9076±0.0114 | 11.380±0.143 | 39.68±0.50 | 636.60±7.98 | 0.6750±0.0085 | 1.5430±0.0193 | 57.990±0.727 | 127.000±1.593 | 889.4±11.2 |
| VN01 | 455.8±5.7 | 413.7±5.2 | 0.6122±0.0077 | 2.655±0.033 | 26.65±0.33 | 402.70±5.05 | 0.3723±0.0047 | 0.4725±0.0059 | 28.680±0.360 | 78.460±0.984 | 522.0±6.5 |
| VN02 | 516.5±6.5 | 929.4±11.7 | 0.6416±0.0080 | 3.851±0.048 | 21.99±0.28 | 472.50±5.93 | 0.3745±0.0047 | 0.4784±0.0060 | 26.850±0.337 | 60.640±0.760 | 452.7±5.7 |
| VN03 | 508.4±6.4 | 400.4±5.0 | 0.5997±0.0075 | 2.416±0.030 | 26.51±0.33 | 474.50±5.95 | 0.3563±0.0045 | 0.4743±0.0059 | 24.040±0.301 | 62.430±0.783 | 414.5±5.2 |
| VN04 | 487.4±6.1 | 573.9±7.2 | 0.6102±0.0077 | 2.937±0.037 | 24.74±0.31 | 444.30±5.57 | 0.3631±0.0046 | 0.4692±0.0059 | 26.190±0.328 | 66.340±0.832 | 457.3±5.7 |
| VN05 | 504.1±6.3 | 634.5±8.0 | 0.6171±0.0077 | 3.068±0.038 | 24.41±0.31 | 463.80±5.82 | 0.3646±0.0046 | 0.4736±0.0059 | 25.690±0.322 | 63.140±0.792 | 441.5±5.5 |
| VN06 | 500.0±6.3 | 536.3±6.7 | 0.6090±0.0076 | 2.807±0.035 | 25.22±0.32 | 460.80±5.78 | 0.3613±0.0045 | 0.4739±0.0059 | 25.310±0.317 | 63.970±0.802 | 437.8±5.5 |
| VN07 | 471.6±5.9 | 487.1±6.1 | 0.6049±0.0076 | 2.676±0.034 | 23.36±0.29 | 423.50±5.31 | 0.3597±0.0045 | 0.4692±0.0059 | 25.120±0.315 | 63.490±0.796 | 435.9±5.5 |
| VN08 | 491.9±6.2 | 552.7±6.9 | 0.6104±0.0077 | 2.850±0.036 | 24.33±0.31 | 449.40±5.64 | 0.3619±0.0045 | 0.4722±0.0059 | 25.370±0.318 | 63.530±0.797 | 438.4±5.5 |
| VN09 | 487.8±6.1 | 525.4±6.6 | 0.6081±0.0076 | 2.778±0.035 | 24.31±0.30 | 444.60±5.58 | 0.3610±0.0045 | 0.4717±0.0059 | 25.270±0.317 | 63.660±0.798 | 437.4±5.5 |
| VN10 | 483.8±6.1 | 521.7±6.5 | 0.6078±0.0076 | 2.768±0.035 | 24.00±0.30 | 439.10±5.51 | 0.3609±0.0045 | 0.4710±0.0059 | 25.250±0.317 | 63.560±0.797 | 437.2±5.5 |
| VN11 | 490.7±6.2 | 557.5±7.0 | 0.6121±0.0077 | 2.881±0.036 | 24.55±0.31 | 447.50±5.61 | 0.3636±0.0046 | 0.4726±0.0059 | 25.780±0.323 | 64.920±0.814 | 447.5±5.6 |
| VN12 | 456.7±5.7 | 511.7±6.4 | 0.5689±0.0071 | 2.654±0.033 | 22.73±0.29 | 416.60±5.22 | 0.3377±0.0042 | 0.4399±0.0055 | 23.780±0.298 | 59.770±0.750 | 411.9±5.2 |
| VN13 | 488.3±6.1 | 530.1±6.6 | 0.6048±0.0076 | 2.783±0.035 | 24.42±0.31 | 446.40±5.60 | 0.3590±0.0045 | 0.4729±0.0059 | 25.180±0.316 | 63.480±0.796 | 436.0±5.5 |
| VN14 | 486.2±6.1 | 543.1±6.8 | 0.6053±0.0076 | 2.820±0.035 | 24.21±0.30 | 443.60±5.56 | 0.3593±0.0045 | 0.4686±0.0059 | 25.290±0.317 | 63.590±0.797 | 438.1±5.5 |
| VN15 | 486.1±6.1 | 540.0±6.8 | 0.6048±0.0076 | 2.809±0.035 | 24.15±0.30 | 443.50±5.56 | 0.3589±0.0045 | 0.4686±0.0059 | 25.200±0.316 | 63.310±0.794 | 436.2±5.5 |
| **Sample** | **^69^Ga** | **^75^As** | **^78^Se** | **^85^Rb** | **^88^Sr** | **^89^Y** | **^90^Zr** | **^93^Nb** | **^95^Mo** | **^103^Rh** | **^137^Ba** |
| Can01 | 9.296±0.117 | 0.3693±0.0046 | 2.6128±0.0328 | 268.54±3.37 | 95.82±1.20 | 0.1372±0.0017 | 0.9769±0.0123 | 0.0267±0.0003 | 4.812±0.060 | 0.0022±0.0000 | 0.0767±0.0010 |
| Can02 | 6.433±0.081 | 0.3333±0.0042 | 2.4294±0.0305 | 269.67±3.38 | 92.71±1.16 | 0.1242±0.0016 | 0.7865±0.0099 | 0.0390±0.0005 | 4.538±0.057 | <MDL | 0.0369±0.0005 |
| Can03 | 10.260±0.129 | 0.3322±0.0042 | 2.2461±0.0282 | 275.41±3.45 | 103.78±1.30 | 0.1078±0.0014 | 0.7735±0.0097 | 0.0276±0.0003 | 5.487±0.069 | 0.0032±0.0000 | 0.0732±0.0009 |
| Can04 | 8.662±0.109 | 0.3450±0.0043 | 2.4294±0.0305 | 271.21±3.40 | 97.44±1.22 | 0.1231±0.0015 | 0.8456±0.0106 | 0.0311±0.0004 | 4.946±0.062 | 0.0018±0.0000 | 0.0622±0.0008 |
| Can05 | 8.450±0.106 | 0.3368±0.0042 | 2.3683±0.0297 | 272.10±3.41 | 97.98±1.23 | 0.1183±0.0015 | 0.8018±0.0101 | 0.0326±0.0004 | 4.990±0.063 | 0.0017±0.0000 | 0.0574±0.0007 |
| Can06 | 8.609±0.108 | 0.3429±0.0043 | 2.4142±0.0303 | 271.43±3.40 | 97.57±1.22 | 0.1219±0.0015 | 0.8347±0.0105 | 0.0315±0.0004 | 4.957±0.062 | 0.0018±0.0000 | 0.0610±0.0008 |
| Can07 | 8.490±0.106 | 0.3384±0.0042 | 2.3800±0.0298 | 271.90±3.41 | 97.88±1.23 | 0.1192±0.0015 | 0.8101±0.0102 | 0.0323±0.0004 | 4.982±0.062 | 0.0017±0.0000 | 0.0583±0.0007 |
| Can08 | 8.964±0.112 | 0.3381±0.0042 | 2.3560±0.0295 | 272.70±3.42 | 99.27±1.24 | 0.1171±0.0015 | 0.8077±0.0101 | 0.0309±0.0004 | 5.101±0.064 | 0.0021±0.0000 | 0.0628±0.0008 |
| US1 | 14.870±0.186 | 0.4709±0.0059 | 1.2680±0.0159 | 276.40±3.47 | 88.11±1.10 | 0.1097±0.0014 | 0.4775±0.0060 | 0.0394±0.0005 | 4.099±0.051 | 0.0022±0.0000 | 0.0744±0.0009 |
| US2 | 14.100±0.177 | 0.3935±0.0049 | 1.4500±0.0182 | 299.70±3.76 | 76.82±0.96 | 0.1627±0.0020 | 0.4555±0.0057 | 0.0384±0.0005 | 6.914±0.087 | 0.0022±0.0000 | 0.0659±0.0008 |
| US3 | 14.480±0.182 | 0.4322±0.0054 | 1.3590±0.0170 | 288.10±3.61 | 82.47±1.03 | 0.1362±0.0017 | 0.4665±0.0058 | 0.0389±0.0005 | 5.507±0.069 | 0.0021±0.0000 | 0.0702±0.0009 |
| US4 | 15.900±0.199 | 0.4745±0.0060 | 1.4920±0.0187 | 316.30±3.97 | 90.53±1.14 | 0.1495±0.0019 | 0.5121±0.0064 | 0.0427±0.0005 | 6.045±0.076 | 0.0024±0.0000 | 0.0770±0.0010 |
| US5 | 14.830±0.186 | 0.4334±0.0054 | 1.4340±0.0180 | 301.40±3.78 | 83.27±1.04 | 0.1494±0.0019 | 0.4780±0.0060 | 0.0400±0.0005 | 6.155±0.077 | 0.0022±0.0000 | 0.0710±0.0009 |
| US6 | 13.130±0.165 | 0.3897±0.0049 | 1.2410±0.0156 | 262.50±3.29 | 74.49±0.93 | 0.1256±0.0016 | 0.4229±0.0053 | 0.0353±0.0004 | 5.105±0.064 | 0.0020±0.0000 | 0.0634±0.0008 |
| US7 | 13.180±0.165 | 0.3845±0.0048 | 1.2790±0.0160 | 268.50±3.37 | 73.93±0.93 | 0.1338±0.0017 | 0.4251±0.0053 | 0.0356±0.0004 | 5.522±0.069 | 0.0020±0.0000 | 0.0631±0.0008 |
| US8 | 15.920±0.200 | 0.4721±0.0059 | 1.5080±0.0189 | 318.90±4.00 | 90.27±1.13 | 0.1531±0.0019 | 0.5130±0.0064 | 0.0428±0.0005 | 6.229±0.078 | 0.0024±0.0000 | 0.0769±0.0010 |
| US9 | 15.820±0.198 | 0.4665±0.0058 | 1.5100±0.0189 | 318.60±4.00 | 89.38±1.12 | 0.1549±0.0019 | 0.5098±0.0064 | 0.0426±0.0005 | 6.333±0.079 | 0.0024±0.0000 | 0.0762±0.0010 |
| Bra1 | 4.487±0.056 | 0.4102±0.0051 | 2.5770±0.0323 | 128.80±1.62 | 133.40±1.67 | 0.2468±0.0031 | 2.2790±0.0286 | 0.0400±0.0005 | 55.440±0.695 | <MDL | <MDL |
| Bra2 | 4.670±0.059 | 0.4231±0.0053 | 2.2150±0.0278 | 111.10±1.39 | 134.50±1.69 | 0.2876±0.0036 | 1.8410±0.0231 | 0.0429±0.0005 | 49.740±0.624 | <MDL | <MDL |
| Bra3 | 4.578±0.057 | 0.4167±0.0052 | 2.3960±0.0300 | 119.90±1.50 | 134.00±1.68 | 0.2672±0.0034 | 2.0600±0.0258 | 0.0414±0.0005 | 52.590±0.659 | <MDL | <MDL |
| Bra4 | 4.578±0.057 | 0.4167±0.0052 | 2.3960±0.0300 | 119.90±1.50 | 134.00±1.68 | 0.2672±0.0034 | 2.0600±0.0258 | 0.0414±0.0005 | 52.590±0.659 | <MDL | <MDL |
| Bra5 | 3.466±0.043 | 0.3154±0.0040 | 1.8130±0.0227 | 90.80±1.14 | 101.40±1.27 | 0.2022±0.0025 | 1.5590±0.0195 | 0.0314±0.0004 | 39.810±0.499 | <MDL | <MDL |
| Bra6 | 4.400±0.055 | 0.4000±0.0050 | 2.2440±0.0281 | 112.40±1.41 | 128.20±1.61 | 0.2606±0.0033 | 1.9130±0.0240 | 0.0400±0.0005 | 49.560±0.621 | <MDL | <MDL |
| VN01 | 18.330±0.230 | 0.1382±0.0017 | 5.3020±0.0665 | 439.50±5.51 | 537.90±6.75 | 0.0724±0.0009 | 0.7463±0.0094 | 0.0751±0.0009 | 4.288±0.054 | <MDL | 0.0765±0.0010 |
| VN02 | 17.980±0.225 | 0.1541±0.0019 | 6.3700±0.0799 | 305.30±3.83 | 542.10±6.80 | 0.0938±0.0012 | 0.7367±0.0092 | 0.1057±0.0013 | 10.120±0.127 | <MDL | 1.1221±0.0141 |
| VN03 | 16.280±0.204 | 0.1354±0.0017 | 5.1030±0.0640 | 384.60±4.82 | 525.30±6.59 | 0.0631±0.0008 | 0.7453±0.0093 | 0.0831±0.0010 | 7.873±0.099 | <MDL | 0.2622±0.0033 |
| VN04 | 17.310±0.217 | 0.1408±0.0018 | 5.5230±0.0693 | 371.80±4.66 | 528.50±6.63 | 0.0755±0.0009 | 0.7336±0.0092 | 0.0869±0.0011 | 7.336±0.092 | <MDL | 0.4809±0.0060 |
| VN05 | 17.190±0.216 | 0.1434±0.0018 | 5.6650±0.0710 | 353.90±4.44 | 532.00±6.67 | 0.0775±0.0010 | 0.7385±0.0093 | 0.0919±0.0012 | 8.444±0.106 | <MDL | 0.6217±0.0078 |
| VN06 | 16.930±0.212 | 0.1399±0.0018 | 5.4300±0.0681 | 370.10±4.64 | 528.60±6.63 | 0.0720±0.0009 | 0.7391±0.0093 | 0.0873±0.0011 | 7.885±0.099 | <MDL | 0.4549±0.0057 |
| VN07 | 16.800±0.211 | 0.1381±0.0017 | 5.3130±0.0666 | 338.60±4.25 | 526.90±6.61 | 0.0693±0.0009 | 0.7336±0.0092 | 0.0810±0.0010 | 5.812±0.073 | <MDL | 0.2787±0.0035 |
| VN08 | 16.970±0.213 | 0.1405±0.0018 | 5.4700±0.0686 | 354.20±4.44 | 529.10±6.63 | 0.0729±0.0009 | 0.7371±0.0092 | 0.0867±0.0011 | 7.380±0.093 | <MDL | 0.4518±0.0057 |
| VN09 | 16.900±0.212 | 0.1395±0.0017 | 5.4040±0.0678 | 354.30±4.44 | 528.20±6.62 | 0.0714±0.0009 | 0.7366±0.0092 | 0.0850±0.0011 | 7.026±0.088 | <MDL | 0.3951±0.0050 |
| VN10 | 16.890±0.212 | 0.1394±0.0017 | 5.3960±0.0677 | 349.00±4.38 | 528.10±6.62 | 0.0712±0.0009 | 0.7357±0.0092 | 0.0842±0.0011 | 6.740±0.085 | <MDL | 0.3752±0.0047 |
| VN11 | 17.160±0.215 | 0.1409±0.0018 | 5.4980±0.0689 | 362.10±4.54 | 530.70±6.65 | 0.0739±0.0009 | 0.7382±0.0093 | 0.0867±0.0011 | 7.291±0.091 | <MDL | 0.4519±0.0057 |
| VN12 | 15.870±0.199 | 0.1309±0.0016 | 5.0950±0.0639 | 332.80±4.17 | 493.40±6.19 | 0.0680±0.0009 | 0.6870±0.0086 | 0.0804±0.0010 | 6.752±0.085 | <MDL | 0.4092±0.0051 |
| VN13 | 16.830±0.211 | 0.1389±0.0017 | 5.3900±0.0676 | 357.20±4.48 | 525.10±6.58 | 0.0715±0.0009 | 0.7325±0.0092 | 0.0853±0.0011 | 7.254±0.091 | <MDL | 0.4182±0.0052 |
| VN14 | 16.880±0.212 | 0.1392±0.0017 | 5.4180±0.0679 | 354.40±4.44 | 525.10±6.58 | 0.0723±0.0009 | 0.7312±0.0092 | 0.0855±0.0011 | 7.192±0.090 | <MDL | 0.4337±0.0054 |
| VN15 | 16.840±0.211 | 0.1391±0.0017 | 5.4080±0.0678 | 352.70±4.42 | 524.70±6.58 | 0.0720±0.0009 | 0.7310±0.0092 | 0.0854±0.0011 | 7.178±0.090 | <MDL | 0.4290±0.0054 |
| **Sample** | **^163^Dy** | **^165^Ho** | **^175^Lu** | **^178^Hf** | **^181^Ta** | **^182^W** | **^185^Re** | **^197^Au** | **^202^Hg** | **^205^Tl** | **^208^Pb** |
| Can01 | <MDL | <MDL | 0.0087±0.0008 | 0.0036±0.0003 | 0.0675±0.0062 | 0.1071±0.0098 | 0.1929±0.0176 | 0.0032±0.0003 | 0.0156±0.0014 | 0.0002±0.0000 | 0.0383±0.0035 |
| Can02 | <MDL | <MDL | 0.0067±0.0006 | 0.0035±0.0003 | 0.0595±0.0054 | 0.1432±0.0131 | 0.2482±0.0226 | 0.0061±0.0006 | 0.0108±0.0010 | 0.0003±0.0000 | 0.0194±0.0018 |
| Can03 | <MDL | <MDL | 0.0077±0.0007 | 0.0035±0.0003 | 0.0510±0.0046 | 0.0947±0.0086 | 0.1147±0.0105 | 0.0051±0.0005 | 0.0167±0.0015 | 0.0003±0.0000 | 0.0250±0.0023 |
| Can04 | <MDL | <MDL | 0.0077±0.0007 | 0.0036±0.0003 | 0.0593±0.0054 | 0.1150±0.0105 | 0.1853±0.0169 | 0.0048±0.0004 | 0.0144±0.0013 | 0.0003±0.0000 | 0.0275±0.0025 |
| Can05 | <MDL | <MDL | 0.0074±0.0007 | 0.0035±0.0003 | 0.0566±0.0052 | 0.1176±0.0107 | 0.1827±0.0167 | 0.0053±0.0005 | 0.0140±0.0013 | 0.0003±0.0000 | 0.0240±0.0022 |
| Can06 | <MDL | <MDL | 0.0076±0.0007 | 0.0036±0.0003 | 0.0586±0.0053 | 0.1156±0.0105 | 0.1846±0.0168 | 0.0049±0.0004 | 0.0143±0.0013 | 0.0003±0.0000 | 0.0266±0.0024 |
| Can07 | <MDL | <MDL | 0.0075±0.0007 | 0.0035±0.0003 | 0.0571±0.0052 | 0.1171±0.0107 | 0.1832±0.0167 | 0.0052±0.0005 | 0.0140±0.0013 | 0.0003±0.0000 | 0.0246±0.0022 |
| Can08 | <MDL | <MDL | 0.0076±0.0007 | 0.0035±0.0003 | 0.0560±0.0051 | 0.1111±0.0101 | 0.1665±0.0152 | 0.0051±0.0005 | 0.0148±0.0013 | 0.0003±0.0000 | 0.0253±0.0023 |
| US1 | <MDL | <MDL | 0.0021±0.0002 | 0.0107±0.0010 | 0.0321±0.0029 | 0.1065±0.0097 | 0.1872±0.0171 | 0.0015±0.0001 | 0.0054±0.0005 | <MDL | 0.0078±0.0007 |
| US2 | <MDL | <MDL | 0.0023±0.0002 | 0.0136±0.0012 | 0.0317±0.0029 | 0.1254±0.0114 | 0.2157±0.0197 | 0.0015±0.0001 | 0.0059±0.0005 | <MDL | 0.0078±0.0007 |
| US3 | <MDL | <MDL | 0.0022±0.0002 | 0.0121±0.0011 | 0.0319±0.0029 | 0.1159±0.0106 | 0.2015±0.0184 | 0.0015±0.0001 | 0.0057±0.0005 | <MDL | 0.0078±0.0007 |
| US4 | <MDL | <MDL | 0.0024±0.0002 | 0.0133±0.0012 | 0.0350±0.0032 | 0.1273±0.0116 | 0.2212±0.0202 | 0.0017±0.0002 | 0.0062±0.0006 | <MDL | 0.0086±0.0008 |
| US5 | <MDL | <MDL | 0.0023±0.0002 | 0.0130±0.0012 | 0.0329±0.0030 | 0.1228±0.0112 | 0.2128±0.0194 | 0.0016±0.0001 | 0.0060±0.0005 | <MDL | 0.0080±0.0007 |
| US6 | <MDL | <MDL | 0.0020±0.0002 | 0.0111±0.0010 | 0.0289±0.0026 | 0.1060±0.0097 | 0.1840±0.0168 | 0.0014±0.0001 | 0.0052±0.0005 | <MDL | 0.0071±0.0006 |
| US7 | <MDL | <MDL | 0.0020±0.0002 | 0.0116±0.0011 | 0.0292±0.0027 | 0.1096±0.0100 | 0.1898±0.0173 | 0.0014±0.0001 | 0.0053±0.0005 | <MDL | 0.0072±0.0007 |
| US8 | <MDL | <MDL | 0.0024±0.0002 | 0.0136±0.0012 | 0.0351±0.0032 | 0.1288±0.0118 | 0.2237±0.0204 | 0.0017±0.0002 | 0.0063±0.0006 | <MDL | 0.0086±0.0008 |
| US9 | <MDL | <MDL | 0.0024±0.0002 | 0.0136±0.0012 | 0.0350±0.0032 | 0.1292±0.0118 | 0.2241±0.0204 | 0.0017±0.0002 | 0.0063±0.0006 | <MDL | 0.0085±0.0008 |
| Bra1 | 0.0103±0.0009 | 0.0024±0.0002 | 0.0043±0.0004 | 0.0073±0.0007 | 0.0874±0.0080 | 0.1520±0.0139 | 0.2867±0.0261 | 0.0608±0.0055 | 0.0166±0.0015 | 0.0021±0.0002 | 0.0238±0.0022 |
| Bra2 | 0.0155±0.0014 | 0.0024±0.0002 | 0.0039±0.0004 | 0.0065±0.0006 | 0.0792±0.0072 | 0.1170±0.0107 | 0.2569±0.0234 | 0.0640±0.0058 | 0.0153±0.0014 | 0.0025±0.0002 | 0.0278±0.0025 |
| Bra3 | 0.0129±0.0012 | 0.0024±0.0002 | 0.0041±0.0004 | 0.0069±0.0006 | 0.0833±0.0076 | 0.1345±0.0123 | 0.2718±0.0248 | 0.0624±0.0057 | 0.0159±0.0015 | 0.0023±0.0002 | 0.0258±0.0024 |
| Bra4 | 0.0129±0.0012 | 0.0024±0.0002 | 0.0041±0.0004 | 0.0069±0.0006 | 0.0833±0.0076 | 0.1345±0.0123 | 0.2718±0.0248 | 0.0624±0.0057 | 0.0159±0.0015 | 0.0023±0.0002 | 0.0258±0.0024 |
| Bra5 | 0.0098±0.0009 | 0.0018±0.0002 | 0.0031±0.0003 | 0.0052±0.0005 | 0.0631±0.0058 | 0.1018±0.0093 | 0.2057±0.0188 | 0.0472±0.0043 | 0.0121±0.0011 | 0.0018±0.0002 | 0.0196±0.0018 |
| Bra6 | 0.0130±0.0012 | 0.0023±0.0002 | 0.0039±0.0004 | 0.0065±0.0006 | 0.0786±0.0072 | 0.1241±0.0113 | 0.2560±0.0234 | 0.0601±0.0055 | 0.0151±0.0014 | 0.0023±0.0002 | 0.0252±0.0023 |
| VN01 | <MDL | <MDL | <MDL | 0.0037±0.0003 | 0.1012±0.0092 | 0.2840±0.0259 | 0.4899±0.0447 | 0.0158±0.0014 | 0.0268±0.0024 | <MDL | 0.0712±0.0065 |
| VN02 | <MDL | 0.0012±0.0001 | 0.0021±0.0002 | 0.0072±0.0007 | 0.0796±0.0073 | 0.1274±0.0116 | 0.2493±0.0227 | 0.0131±0.0012 | 0.0255±0.0023 | <MDL | 0.0648±0.0059 |
| VN03 | <MDL | <MDL | <MDL | <MDL | 0.0680±0.0062 | 0.1122±0.0102 | 0.2300±0.0210 | 0.0146±0.0013 | 0.0219±0.0020 | <MDL | 0.0197±0.0018 |
| VN04 | <MDL | 0.0004±0.0000 | 0.0007±0.0001 | 0.0036±0.0003 | 0.0819±0.0075 | 0.1724±0.0157 | 0.3191±0.0291 | 0.0143±0.0013 | 0.0244±0.0022 | <MDL | 0.0512±0.0047 |
| VN05 | <MDL | 0.0005±0.0000 | 0.0009±0.0001 | 0.0036±0.0003 | 0.0765±0.0070 | 0.1373±0.0125 | 0.2661±0.0243 | 0.0140±0.0013 | 0.0239±0.0022 | <MDL | 0.0452±0.0041 |
| VN06 | <MDL | 0.0003±0.0000 | 0.0006±0.0001 | 0.0024±0.0002 | 0.0754±0.0069 | 0.1406±0.0128 | 0.2717±0.0248 | 0.0143±0.0013 | 0.0234±0.0021 | <MDL | 0.0387±0.0035 |
| VN07 | <MDL | 0.0002±0.0000 | 0.0004±0.0000 | 0.0018±0.0002 | 0.0749±0.0068 | 0.1423±0.0130 | 0.2745±0.0250 | 0.0137±0.0012 | 0.0231±0.0021 | <MDL | 0.0355±0.0032 |
| VN08 | <MDL | 0.0003±0.0000 | 0.0006±0.0001 | 0.0026±0.0002 | 0.0756±0.0069 | 0.1401±0.0128 | 0.2708±0.0247 | 0.0140±0.0013 | 0.0235±0.0021 | <MDL | 0.0398±0.0036 |
| VN09 | <MDL | 0.0003±0.0000 | 0.0005±0.0000 | 0.0023±0.0002 | 0.0753±0.0069 | 0.1410±0.0129 | 0.2724±0.0248 | 0.0140±0.0013 | 0.0233±0.0021 | <MDL | 0.0380±0.0035 |
| VN10 | <MDL | 0.0003±0.0000 | 0.0005±0.0000 | 0.0022±0.0002 | 0.0753±0.0069 | 0.1411±0.0129 | 0.2726±0.0249 | 0.0139±0.0013 | 0.0233±0.0021 | <MDL | 0.0377±0.0034 |
| VN11 | <MDL | 0.0003±0.0000 | 0.0006±0.0001 | 0.0029±0.0003 | 0.0784±0.0071 | 0.1538±0.0140 | 0.2916±0.0266 | 0.0142±0.0013 | 0.0239±0.0022 | <MDL | 0.0442±0.0040 |
| VN12 | <MDL | 0.0003±0.0000 | 0.0006±0.0001 | 0.0025±0.0002 | 0.0715±0.0065 | 0.1363±0.0124 | 0.2610±0.0238 | 0.0131±0.0012 | 0.0220±0.0020 | <MDL | 0.0385±0.0035 |
| VN13 | <MDL | 0.0003±0.0000 | 0.0005±0.0000 | 0.0024±0.0002 | 0.0753±0.0069 | 0.1417±0.0129 | 0.2730±0.0249 | 0.0140±0.0013 | 0.0233±0.0021 | <MDL | 0.0389±0.0035 |
| VN14 | <MDL | 0.0003±0.0000 | 0.0006±0.0001 | 0.0026±0.0002 | 0.0760±0.0069 | 0.1447±0.0132 | 0.2773±0.0253 | 0.0140±0.0013 | 0.0234±0.0021 | <MDL | 0.0408±0.0037 |
| VN15 | <MDL | 0.0003±0.0000 | 0.0006±0.0001 | 0.0025±0.0002 | 0.0754±0.0069 | 0.1419±0.0129 | 0.2731±0.0249 | 0.0139±0.0013 | 0.0233±0.0021 | <MDL | 0.0397±0.0036 |
